# Supplementary material for: Direct prediction of regulatory elements from partial data without imputation
Source: PLoS Comput Biol. 2019 Nov 4;15(11):e1007399. doi: 10.1371/journal.pcbi.1007399 (PMC6855516; doi:10.1371/journal.pcbi.1007399)
Supplement: S1 Table — (DOCX) [file pcbi.1007399.s001.docx]

**Supplemental Table 1:** Mean R^2^ of gene expression prediction using both TSS and distal regions.

|  | **5 marks**  (fixed to 42 states) | **5 marks** (20 states) | **12 marks** (42 states) |
| --- | --- | --- | --- |
| *Low mean, low std. dev. genes* | 7.6% | 9.0% | 9.2% |
| *Low mean, high std. dev. genes* | 19.5% | 24.9% | 25.3% |
| *High mean, high std. dev. genes* | 29.5% | 37.3% | 40.1% |
| *High mean, low std. dev. genes* | 10.2% | 11.2% | 13.8% |
