# Supplementary material for: Direct prediction of regulatory elements from partial data without imputation
Source: PLoS Comput Biol. 2019 Nov 4;15(11):e1007399. doi: 10.1371/journal.pcbi.1007399 (PMC6855516; doi:10.1371/journal.pcbi.1007399)
Supplement: S2 Table — (DOCX) [file pcbi.1007399.s002.docx]

**Supplemental Table 2:** Mean R^2^ of eRNA prediction.

|  | **5 marks**  (fixed to 42 states) | **5 marks** (20 states) | **12 marks** (42 states) |
| --- | --- | --- | --- |
| *Fantom5 eRNA TPM* | 22.0% | 25.1% | 27.3% |
| *Fantom5 eRNA peaks* | 14.1% | 17.1% | 19.2% |
