# Supplementary material for: Direct prediction of regulatory elements from partial data without imputation
Source: PLoS Comput Biol. 2019 Nov 4;15(11):e1007399. doi: 10.1371/journal.pcbi.1007399 (PMC6855516; doi:10.1371/journal.pcbi.1007399)
Supplement: Attachment — Submitted filename: reviewer-response.20190822.docx [file pcbi.1007399.s005.docx]

**Direct prediction of regulatory elements from partial data without imputation**

Yu Zhang & Shaun Mahony

**PCOMPBIOL-D-19-00827.R2**

**Responses to Reviewer Comments**

We thank all three reviewers for their insightful evaluation of our work. Reviewers 1 & 2 had no further comments in this round of review. Our responses to Reviewer 3’s comments are provided in blue below.

**Reviewer 3**

The addition of two different sections and some text to clarify limitations (I appreciate the truthful statement about the imputation part in the Discussion) have strengthened the manuscript. There are two points that are not fully addressed either because of time limitation or misunderstanding of my suggestions, which would be great to do so before publication.

- For my comment on using only previous iteration of IDEAS results as gold standard, for the completeness of this manuscript, that would be important to add a summary of the comparative results of IDEAS and other methods from the previous work. That would justify, to some extent, the use of IDEAS-only evaluation together with the added text.

We agree with the reviewer regarding the value of adding a justification for our focus on the IDEAS method and variants thereof. We have therefore added the following text to p.6:

“The focus of this study is to evaluate the power of including partially available data in segmentation. We therefore compared our proposed approach with several other approaches to handling partial data based on a single segmentation algorithm so that differences between segmentation algorithms will not confound the comparison results. We choose to use the IDEAS method as the basis for comparison for three reasons: 1) our previous studies [17,18] have shown uniformly superior power of IDEAS over existing segmentation tools on predicting a wide variety of genomic features, such as gene expression, DNA methylation, annotated enhancers, GWAS variants, eQTLs, sequence-derived functional scores, and chromatin interaction data; 2) the proposed direct imputation method on partial data is feasible only within the IDEAS framework; and 3) the concatenation approach used in our simulation study can be treated as a proxy for the other segmentation algorithms that do not take positional information into account.”

- The comment on "why more states are better" was mainly referring to a controlled experiment where the number of states are set to an equal number for both the complete 5-mark segmentation and the incomplete 12-mark. The evaluations that are done would still be applicable but differences could then directly be attributed to using more marks including those that are complete rather than, say, more granular state assignments due to simply having more marks (i.e., 5 marks are not rich enough to have 42 meaningfully distinct states but 12 with imputation are).

We assessed the performance of a fixed-42-state model, trained using the 5 common marks, as suggested by the reviewer. We describe the results of this new analysis on p.16, as follows:

“Since the number of states are different between the two segmentations, we performed an additional experiment to check if the improved performance of the 12-mark segmentation is merely a result of its increased number of states. We ran IDEAS on the 5 common marks, but constrained the model to infer 42 states, thereby matching the number of states in the partial 12-mark segmentation. We used the resulting segmentation to predict RNA levels and Fantom5 eRNAs as before. Consistently in all comparisons (Suppl Table 1 & Suppl Table 2), we observed inferior predictive power compared with both the original 20-state (5 common mark) model and the new 42-state (partial 12-mark) model. These results suggest that the improved predictive power of the new segmentation is attributable to the additional marks used.”

**Have all data underlying the figures and results presented in the manuscript been provided?**

...

Reviewer #3: No: Supplementary tables need to be provided with segmentation information from each dataset for reproduction of at least each main figure.

We have provided URLs to the code (GitHub) and UCSC browser tracks for the ROADMAP segmentation results (hg19 & hg38). We performed a large number of simulated missing data permutations to create Figures 2-4, and thus we created a large number of segmentation tracks for those figures. Since our approach is reproducible from our code and described methods, we respectfully disagree that it will be of value to store and provide web hosting for this large number of segmentations.

**Additional changes**

- We have added Supplementary Figures 1 & 2. These figures extend from Figure 4 (“Comparison of accuracy of imputed chromatin marks between IDEAS and ChromImpute”). Whereas Figure 4 only shows results for the 8 missing marks simulations, the new supplementary figures show results for 10 and 11 missing marks. In the previous version of our manuscript, we stated: “Results for 10 or 11 missing marks are similar (data not shown)”. The additional supplementary figures remove the need for the “data not show” statement.
- We have edited Figure 5 to show a better example that illustrates how IDEAS using 12 incomplete marks yields more informative segmentations compared with IDEAS using 5 complete marks.
- We have added an Author Summary section as required by the manuscript submission guidelines.
